# Autoimmune Thyroid Disorders

**DOI:** 10.4061/2011/432890

**Published:** 2011-12-29

**Authors:** Rosalind Brown, Gary L. Francis

**Affiliations:** ^1^Pediatrics, Harvard Medical School, Boston, MA 02115, USA; ^2^Section of Pediatric Endocrinology and Metabolism, Children's Hospital of Richmond at the Virginia Commonwealth University Health System, P. O. Box 980140, Richmond, VA 23098, USA

Thyroid autoimmunity, as reflected by the presence in serum of autoantibodies directed against the thyroid autoantigens thyroglobulin (Tg) and thyroid peroxidase (TPO), is present in >10% of the US population over 12 years of age [1] and is the most common cause of endocrine dysfunction in iodine-sufficient populations [2]. The underlying mechanism is a failure of T-cell tolerance leading to lymphocytic infiltration of the thyroid gland [3] and to a complex sequence of humoral and cellular immune responses to thyroid antigens, presumably in response to an environmental trigger [4]. In chronic lymphocytic thyroiditis (CLT), the predominant immunologic mechanisms are T-cell- and cytokine-mediated thyroid cell damage and apoptotic cell death whereas in Graves' disease (GD) generation of thyrotropin (TSH) receptor autoantibodies leads to thyroid cell stimulation [5], but significant overlap exists. Seven susceptibility genes, in addition to the major histocompatibility gene (HLA-DR3), have now been identified [6]. Some of these genes affect the immune response in general (CD40, CTLA-4, and PTPN22), while others are thyroid specific (thyroglobulin, thyrotropin (TSH) receptor). Some are common to both CLT and GD, while others are specific for GD.

 In view of the importance of AITD as well as the diverse array of new information, it is only fitting that this special issue of the Journal of Thyroid Research is devoted entirely to this complex subject. Four of the papers we have selected are focused on clinical topics, including AITD in childhood, during pregnancy, in the postpartum period, and in patients with type 1 diabetes mellitus. The fifth paper addresses the potential role of NKT cells in an animal model of thyroiditis.

We conclude this special edition with a discussion of thyroid autoimmunity in patients with papillary thyroid cancer (PTC). The association of AI and thyroid cancer was first reported by Dailey et al. [7]. In general, patients with AI appear more likely to have PTC than follicular thyroid cancer (FTC), but a lower frequency of extrathyroidal extension, nodal and distant metastases when compared with patients without AI. In some but not all series, patients with autoimmune thyroiditis (AT) and PTC have improved survival when compared to those with PTC alone, suggesting that thyroid autoimmunity might contribute to improved survival [8–10]. In contrast, other data suggest that AI might actually increase the risk to develop thyroid cancer [10–12].

Several theories have been proposed to explain how AI might increase the risk for thyroid malignancy. Thyrocyte apoptosis and proliferation are increased in AI suggesting that thyrocytes rapidly progressing through the cell cycle might accumulate increased DNA damage resulting in malignant transformation [13]. Russell et al. hypothesized that thyroid cells predestined to become cancers might secrete proinflammatory cytokines that affect immune cells [14]. They showed that thyrocytes of ret/PTC3 transgenic mice express increased levels of interleukins, tumor necrosis factor-*α*, and cyclooxygenase-2 [14] that could attract and/or activate cells of the immune system. Finally, the ret/PTC recombinant genes have been detected in samples of AI [15–17] suggesting that ret/PTC rearrangements might be present in AI and could be precursors to PTC.

 From these papers, it is clear that thyroid autoimmunity is a frequent problem in the population and that thyroid autoimmunity can lead to a variety of thyroid disorders including alterations in thyroid hormone synthesis and possibly even neoplasia. Focused research in this area is beginning to illuminate some of the molecular mechanisms that help to explain these associations.

*Rosalind Brown* *Rosalind Brown* *Gary L. Francis* *Gary L. Francis*
